# Identification and Elimination of Antifungal Tolerance in *Candida auris*

**DOI:** 10.3390/biomedicines11030898

**Published:** 2023-03-14

**Authors:** Samira Rasouli Koohi, Shamanth A. Shankarnarayan, Clare Maristela Galon, Daniel A. Charlebois

**Affiliations:** 1 Department of Physics, University of Alberta, Edmonton, AB T6G 2R3, Canada; 2 Department of Biological Sciences, University of Alberta, Edmonton, AB T6G 2E9, Canada

**Keywords:** adjuvant, antifungal tolerance/resistance, broth microdilution assay, *Candida auris*, disk diffusion assay, diskImageR, human fungal pathogen

## Abstract

Antimicrobial resistance is a global health crisis to which pathogenic fungi make a substantial contribution. The human fungal pathogen *C. auris* is of particular concern due to its rapid spread across the world and its evolution of multidrug resistance. Fluconazole failure in *C. auris* has been recently attributed to antifungal “tolerance”. Tolerance is a phenomenon whereby a slow-growing subpopulation of tolerant cells, which are genetically identical to susceptible cells, emerges during drug treatment. We use microbroth dilution and disk diffusion assays, together with image analysis, to investigate antifungal tolerance in *C. auris* to all three classes of antifungal drugs used to treat invasive candidiasis. We find that (1) *C. auris* is tolerant to several common fungistatic and fungicidal drugs, which in some cases can be detected after 24 h, as well as after 48 h, of antifungal drug exposure; (2) the tolerant phenotype reverts to the susceptible phenotype in *C. auris*; and (3) combining azole, polyene, and echinocandin antifungal drugs with the adjuvant chloroquine in some cases reduces or eliminates tolerance and resistance in patient-derived *C. auris* isolates. These results suggest that tolerance contributes to treatment failure in *C. auris* infections for a broad range of antifungal drugs, and that antifungal adjuvants may improve treatment outcomes for patients infected with antifungal-tolerant or antifungal-resistant fungal pathogens.

## 1. Introduction

Antimicrobial resistance (AMR) threatens the advances of modern medicine. Antifungal resistance contributes significantly to the AMR problem [1,2], especially among immunocompromised patients [3,4]. A multitude of biological, sociological, and economic factors result in hundreds of millions of serious fungal infections and between 1 and 1.5 million fungal infection-related deaths per year globally [5,6]. AMR, among fungi, is of particular concern due to the limited number of classes of drugs available to treat invasive fungal infections (i.e., fungistatic azoles as well as fungicidal polyenes and echinocandins) [7]. This threat is exacerbated by the fact that no new class of antifungal drugs has reached the market in over a decade [8,9]. Climate change is also predicted to increase the prevalence of fungal infections, as fungi adapt to warmer temperatures to increase their geographic range and overcome the thermal protection barrier of their warm-blooded hosts [10].

*Candida* species of yeast are the most common causes of fungal infections [11]. One *Candida* species that is increasingly of concern is *Candida auris* [12], due to its resistance to antifungal drugs and healthcare-associated outbreaks [13]. *C. auris* has now been reported on all inhabited continents and in over 47 countries [14,15]. Particularly concerning, is that *C. auris* is multidrug resistant (i.e., non-susceptible to at least one agent in three or more classes of antimicrobials) [16,17,18], and, in some cases, it has been found to be pandrug-resistant (i.e., non-susceptible to all agents in all antimicrobial classes) [18,19]. *C. auris* has mortality rates of up to 45% among patients with bloodstream infections [20].

“Tolerance” is a phenomenon whereby a slow-growing subpopulation of cells, which are thought to be genetically identical to susceptible cells, emerges during antifungal drug treatment [21]. Antifungal tolerance is distinct from antifungal resistance, in that resistance is the result of heritable genetic changes and resistant cells grow above the minimum inhibitory concentration (MIC) in a concentration-dependent manner (i.e., MIC increases in resistance, but it does not increase in tolerance). In contrast, tolerance is a reversible phenomenon whereby cells grow slowly above MIC (i.e., they exhibit growth at “supra-MIC”). Tolerance manifests from the phenotypic heterogeneity intrinsic to a given fungal isolate, such that any cell within an isogenic population can reproduce the fractions of susceptible and tolerant cells present prior to the initiation of antifungal treatment. Cross tolerance has been observed in *C. albicans*, whereby strains tolerant to posaconazole also exhibit tolerance to other azole drugs [22]. Though the molecular mechanisms underlying tolerance in *Candida* species are still largely unknown, preliminary studies have shown that tolerance is associated with multiple genetic components that differ between isolates, including Hsp90-faciliated azole tolerance in *C. auris* [23]. Aneuploidy has also been shown to alter antifungal tolerance in *C. albicans* [24,25]. It is unknown if *C. auris* is tolerant to non-azole classes of antifungal drugs.

Clinical assays have not been designed to detect antifungal tolerance [26,27]. Quantitatively measuring tolerances of infecting isolates may provide prognostic insights concerning the success of mono- and combination-antifungal therapies [28]. Broth microdilution assays and disk diffusion assays, coupled with the image analysis software diskimageR, have been successfully used to quantify antifungal tolerance in research laboratories [29]. Most clinical diagnostic tests are performed on cultures grown for 24 h and therefore cannot detect drug-tolerant cells, which are typically visually evident after 48 h of growth [21]. Tolerance, along with host factors, immune status, and pharmacological issues [30], may explain why some patients do not respond to drug therapy despite being infected with fungi that have been determined, by traditional antimicrobial susceptibility testing methods, to be susceptible to a particular drug (i.e., cells that do not grow above MIC at 24 h, the standard endpoint MIC measurement for *Candida* species) [21,28]. “Trailing growth” (the clinical term for tolerance) leads to poor response to fluconazole in *C. tropicalis* in wax moth larvae [31] and mouse models [32], and high levels of tolerance are associated with *C. albicans* infections in patients treated with fluconazole [33].

Adjuvant drugs have the potential to sustain the vital functions of antimicrobial drugs [21]. Non-antifungal agents have been shown to enhance the effectiveness of azole drugs against resistant *Candida* species and other pathogenic fungi, including *Aspergillus fumigatus*, *Cryptococcus neoformans*, and the dimorphic fungus *Histoplasma capsulatum* [34,35,36]. Specifically, the antimalarial drug chloroquine, in combination with fluconazole, exhibited enhanced antifungal activity against *C. albicans*, *C. tropicalis*, *C. glabrata, C. parapsilosis*, and *C. krusei* (teleomorph is known as *Issatchenkia orientalis* and *Pichia kudriavzevii* [11]) isolates in vitro [37]. Whether or not tolerance and resistance to azoles or to other classes of antifungal drugs can be eliminated in *C. auris* using adjuvant antifungal therapies, remains to be investigated. Another study explored the activity of doxycycline, pyrvinium pamoate, along with chloroquine, as adjuvants in combination with fluconazole in clinical *C. albicans* isolates, and found increased antifungal activity [29]. Chloroquine is a member of the quinoline family and is used to treat diseases including malaria, amebiasis, rheumatoid arthritis, discoid, and systemic lupus erythematosus [38,39,40]. Chloroquine causes iron depletion, leading to a decrease in membrane sterol availability and downregulates the *ERG11* gene [41]. We hypothesize that the combining chloroquine with common antifungal drugs will eliminate antifungal tolerance in *C. auris*.

The main aims of our study are to use broth microdilution and disk diffusion assays, together with diskImageR, to investigate if tolerance to all three classes of antifungal drugs occurs in *C. auris,* and if this tolerance can be eliminated by adjuvant antifungal therapy. We find that *C. auris* is tolerant to several fungistatic and fungicidal drugs: fluconazole, itraconazole, posaconazole, voriconazole, amphotericin B, and caspofungin. We demonstrate that antifungal tolerance is detectable at 24 h, as well as at 48 h, and that tolerance is a reversible phenomenon. Finally, we are reporting for the first time that in some isolates combining antifungal drugs with the adjuvant chloroquine eliminates tolerance and resistance in *C. auris*.

## 2. Materials and Methods

### 2.1. Strains, Media, and Growth Conditions

*C. auris* isolates were obtained from clinical samples from the Alberta Precision Laboratories (APL)—Public Health Laboratory (ProvLab).

All strains and isolates (Table S1) were preserved in 25% glycerol at −80 °C until further use. The strains and isolates were revived by culturing from frozen stock on YPD agar plates (yeast extract: Sigma Aldrich, #8013-01-2; bacto peptone: Difco, #9295043) and incubated at 35 °C for 48 h. Fresh subcultures were made on YPD agar plates and incubated at 35 °C for 24 h prior to conducting microbroth dilution and disk diffusion assays (Section 2.3). 

### 2.2. DNA Extractions, PCR, and Sequencing

The initial identification of all *C. auris* isolates was performed using matrix-assisted laser desorption ionization—time of flight (MALDI-TOF) mass spectrometry [42,43] by the APL—ProvLab. The molecular identity of these isolates was confirmed by amplifying and sequencing the Internal Transcribed Spacer (ITS) region of ribosomal DNA. The primers ITS-5 (5′-GGAAGTAAAAGTCGTAACAAGG-3′) and ITS-4 (5′-TCCTCCGCTTATTGATATGC-3′) were used to amplify the ITS region (Integrated DNA Technologies). Genomic DNA was extracted using manual phenol–chloroform–isoamyl alcohol method [44]. The concentration of the extracted DNA was measured using a microvolume μDrop Plate (Thermo Fisher Scientific, Mississauga, ON, Canada, #N12391). The template and the primers were mixed in concentrations of 7.5 ng/μL and 0.25 μM, respectively, to a final volume of 10 μL. Sanger sequencing was then performed using a 3730 Genetic Analyzer (Thermo Fisher Scientific, Mississauga, ON, Canada, #A41046) at the Molecular Biology Services Unit at the University of Alberta. The resulting sequences were subjected to nucleotide BLAST analysis [45], which revealed 100% similarly to the standard strains. The *C. auris* isolates’ ITS sequences were submitted to NCBI with the accession number OP984814-OP984818.

### 2.3. Broth Microdilution and Disk Diffusion Assays

The MIC for each isolate was first determined via broth microdilution assays following CLSI M27 guidelines [46]. All the isolates were tested in 96-well U-bottom microwell plates (Thermo Fisher Scientific, Mississauga, ON Canada, #163320) against fluconazole (Sigma-Aldrich, Oakville, ON, Canada, #F8929) (0.12–64 µg/mL), amphotericin B (Sigma-Aldrich, Canada, #A9528) (0.03–16 µg/mL), itraconazole (Sigma-Aldrich, Oakville, ON, Canada, #16657) (0.03–16 µg/mL), posaconazole (Sigma-Aldrich, Oakville, ON, Canada, #SML2287) (0.03–16 µg/mL), voriconazole (Sigma-Aldrich, Oakville, ON Canada, #P20005) (0.03–16 µg/mL), micafungin (Sigma-Aldrich, Oakville, ON, Canada, #208538) (0.015–8 µg/mL), caspofungin (Sigma-Aldrich, Oakville, ON, Canada, #179463-17-3) (0.015–8 µg/mL), and anidulafungin (Sigma-Aldrich, Oakville, ON, Canada, #166663-25-8) (0.03–16 µg/mL). These antifungals were dissolved in DMSO (fluconazole, itraconazole, voriconazole, posaconazole, anidulafungin, and amphotericin B) or water (caspofungin and micafungin); the concentration of the antifungal microwell plates were twice the final concentration tested with the inoculum added. Freshly cultured *Candida* species (*C. auris*, *C. parapsilosis* (ATCC 22019), and *I. orientalis* (ATCC 6258)) at 24 h of incubation at 35 °C were used as inoculum. Inoculum of 100 µL consisting of 2–5 × 10^3^ cells were used to inoculate the antifungal microwell plates. After inoculation, the microwell plates were incubated at 35 °C and evaluated after 24 h and 48 h to determine the MICs.

Disk diffusion assays (DDAs) were carried out as per CLSI M44-A2 guidelines [47] against fluconazole (25 µg), itraconazole (50 µg), posaconazole (5 µg), voriconazole (1 µg), amphotericin B (20 µg), and caspofungin (5 µg). MHA medium with 2% dextrose (Sigma Aldrich, Oakville, ON, Canada, #50-99-7) and 0.5 μg/mL methylene blue dye (Sigma Aldrich, Oakville, ON, Canada, #03978) was used to perform the disk diffusion assays. After 24 h of growth, 5–10 colonies were picked and liquid suspensions of *C. auris* were made by reconstituting colonies in 2 mL of normal saline (Sigma Aldrich, Oakville, ON, Canada, #S8776). The optical density (OD) was measured using a Varioskan LUX microplate reader (Thermo Fisher Scientific, Mississauga, ON, Canada, #N16044) at 530 nm, and adjusted to an OD of 0.09–0.13, which corresponded to 1–5 × 10^6^ cells/mL. The adjusted solution was utilized to swab on the Muller–Hinton agar (MHA) using sterile cotton swabs (Fisher Scientific, Saint-Laurent, Quebec, Canada, #22-029-683). An antifungal disk was placed on each plate after inoculating and drying the agar plates. The plates were then incubated for 24 to 48 h at 35 °C. All experiments were performed in triplicate.

### 2.4. Photography and Image Preprocessing

Photographs of each disk diffusion plate were taken after 24 h and 48 h at the maximum possible resolution (6000 by 4000 pixels with an aspect ratio of 3:2) using a Canon EOS Rebel SL3 camera with a Canon EF-S 35 mm f/2.8 Macro IS STM macro lens. The camera settings were as follows: ISO 800, white balance, picture type “neutral”, time 1/100 s, center focused against a plain black background from a fixed distance. The photos were taken and then the size of each photograph was standardized by cropping the edges and bringing all images to the same resolution.

### 2.5. Quantifying Tolerance via Supra-MIC Growth and Fraction of Growth

Tolerant subpopulations grow slowly in drug concentrations above MIC [21]. We used established methods to quantify tolerance, namely, supra-MIC growth from microbroth dilution assays and the fraction of growth (FoG) in the zone of inhibition (ZOI) from disk diffusion assays (Section 2.3). 

The MIC for each isolate was determined using CLSI supplement M60 guidelines [48]. The MIC readings were recorded at 24 h and 48 h post inoculation. Tentative breakpoints provided by the Centers for Disease Control and Prevention for *C. auris* were considered to differentiate them as susceptible or resistant [49]. *I. orientalis* and *C. parapsilosis* were used as reference strains to ensure that the antifungal MIC range in each experiment was within CLSI guidelines.

Supra-MIC growth (*SMG*) was determined by subjecting the antifungal microwell plates used for measuring MICs to spectrophotometric reading at 630 nm after 24 h and 48 h of incubation at 35 °C. *SMG* was calculated as an average growth per well above MIC-normalized to total growth without antifungals [28]:

(1) $$SMG=\frac{average\;growth\;per\;well\;above\;MIC}{growth\;without\;antifungal}$$

The software program diskImageR [29] analyses photographs of disk diffusions assays. diskImageR utilizes the image processing program ImageJ [50] and the programming language *R* [51]. We used diskImageR to measure the tolerance and resistance of *C. auris* isolates to fungistatic and fungicidal drugs from photographs of the disk diffusion assay plates (Section 2.4; Figures S1 and S2). All disk diffusion experiments were repeated in triplicate using antifungal disks placed in the center of MHA plates incubated at 35 °C for 24 and 48 h (Figure S3). After the photographs were imported by diskImageR into ImageJ, the size of each photograph was standardized and the “find particles” macro was used to find the center of the antimicrobial diffusion disk. The radius of the ZOI (RAD) and the FoG in the ZOI were measured where 20%, 50%, and 80% of growth was inhibited (RAD_20_, RAD_50_, and RAD_80_, and FoG_20_, FoG_50_, and FoG_80_, respectively). The RAD measures the degree of susceptibility/resistance, and FoG measures the degree of tolerance. The RAD for all disk diffusions assay plates were also measured manually (using a ruler), and the FoGs were also analyzed using ImageJ [52]. ImageJ analysis for estimating pixel intensity to obtain FoG was carried out by importing photographs to ImageJ software and setting “on” the measurements such as “mean grey value”, minimum and maximum grey “area”, and fixing the “area” for ZOI. The “measure” macro was then used to measure the pixel intensity. For photographs of 48 h DDA plates, the same parameters were restored to their 24 h counterparts, and the pixel intensity was measured within ZOI. When there are colonies at border of the ZOI (e.g., Figure S3B), diskImageR considers it as the area outside of the ZOI, and the measured RAD is smaller than the manually measured RAD; consequently, the FoG_20_ measured by diskImageR is also inaccurate. Therefore, in these cases, the RAD was obtained by manually measuring the RAD and by measuring the FoG using ImageJ (Figure S4) [50]. When isolates were highly tolerant, resulting in many colonies in the ZOI (Figure S5B) or complete confluence in the ZOI (Figure S5D) after 48 h, diskImageR reported RAD and FoG as “NA” (Not Applicable). 

### 2.6. Experiments to Determine Effectiveness Adjuvant-Antifungal Treatment 

The synergies among antifungals (fluconazole, itraconazole, posaconazole, voriconazole, amphotericin B, and caspofungin) and adjuvant (chloroquine) against *C. auris*, *C. parapsilosis*, and *I. orientalis* were evaluated using DDAs (Section 2.3) and broth microdilution methods with minor modifications. For DDAs, a syringe-filtered chloroquine diphosphate salt (Sigma-Aldrich, #C6628) solution was added to MHA media after autoclaving to a final concentration of 1031.8 µg/mL. After inoculation of *C. auris* and the control strains, the MHA plates containing chloroquine were incubated in the dark as chloroquine light sensitive. These plates were read and photographed at 24 h and 48 h. *C. auris* isolates and control strains were lawn cultured (i.e., the entire surface of the agar plate was covered by swabs dipped in the liquid culture) on the MHA plates containing chloroquine with and without antifungal disks, to respectively determine the effect of antifungal chloroquine and chloroquine alone on *C. auris*. Whereas for the broth microdilution method, the concentration for different antifungal drugs were as mentioned in Section 2.3 and the chloroquine concentration ranged from 8 to 512 µg/mL. Synergistic activity of chloroquine with different antifungals was tested using the checkerboard method as previously described [37]. Both antifungal drugs (50 µL) and chloroquine (50 µL) were dispensed to sterile 96 well U bottom microtiter plates and prepared inoculum (100 µL) as per Section 2.3 was inoculated. Plates were then incubated at 35 °C. MIC and SMG results were read at 24 h and 48 h.

## 3. Results

### 3.1. Identification of Resistance in C. auris from Broth Microdilution Assays

**Figure 1:**
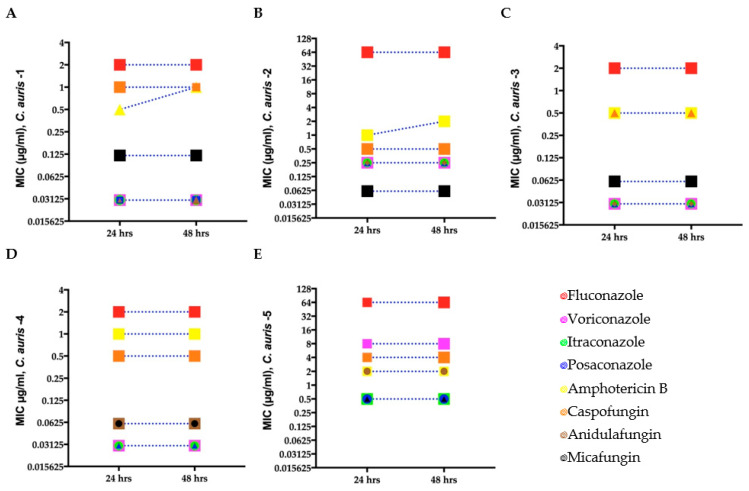
Minimum inhibitory concentration (MIC) for clinical *C. auris* isolates (**A**–**E**) growing in antifungal microwell plates to determine susceptibility/resistance to antifungal drugs. Mean MICs of five clinical *C. auris* isolates measured after 24 and 48 h for four fungistatic drugs (fluconazole, itraconazole, posaconazole, and voriconazole) and two fungicidal drugs (amphotericin B and caspofungin). Different symbols denote *C. auris* isolates with the same MIC.

To determine if the *C. auris* isolates were resistant to the antifungal drugs used in our study, we performed antifungal susceptibility testing at 24 and 48 h using the broth microdilution method (Section 2.3). The MICs for the *C. auris* isolates indicated that three isolates were susceptible to the fungicidal and fungistatic drug tested, whereas *C. auris* isolate 2 was not susceptible to fluconazole, and *C. auris* isolate 5 was not susceptible to fluconazole, voriconazole, caspofungin, and amphotericin B (Figure 1 and Table S2). The quality control strains *C. parapsilosis* and *I. orientalis* were within the recommended ranges. No change in MIC was observed at 24 and 48 h except for *C. auris* isolates 1 and 2 against amphotericin B.

### 3.2. Identification of Resistance in C. auris from Disk Diffusion Assays

**Figure 2:**
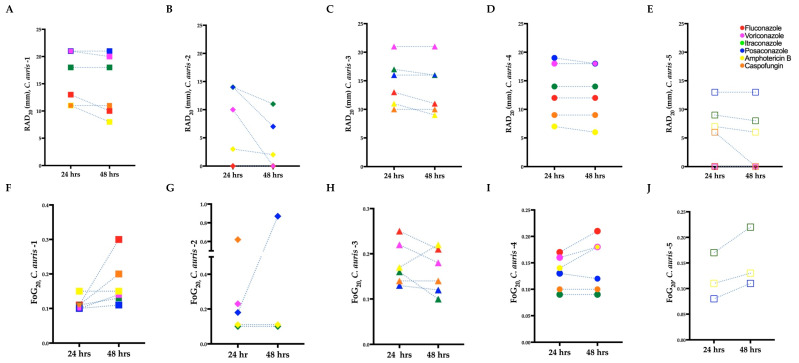
Radius of the zone of inhibition (RAD) (**A**–**E**) and fraction of growth in the zone of inhibition (FoG_20_) (**F**–**J**) for *C. auris* isolates treated with antifungal drugs. Mean RAD where 20% of growth is inhibited (RAD_20_) at 24 and 48 h. (**B**) Mean FoG where 20% of growth is inhibited (FoG_20_) 24 and 48 h. *C. auris* isolate 2 treated with caspofungin at 48 h is not plotted in (**B**), as it exhibited FoG in the entire ZOI (i.e., a “NA” data point was generated by diskImageR [29]); the reduction in RAD and FoG_20_ for *C. auris* isolate 3 in (**C**) and (**H**), respectively, is due to the exclusion of FoG_20_ within the ZOI by diskImageR (see Section 2.5 for details). *C. auris* isolate 5 exhibited resistances to fluconazole and caspofungin.

To confirm the resistance of the *C. auris* isolates determined by the broth microdilution assays (Section 3.1), we performed the corresponding disk diffusion assays. In agreement with the microbroth dilution method, resistance was noted in *C. auris* isolate 2 for fluconazole and *C. auris* isolate 5 for fluconazole and voriconazole (RAD = 0 mm in all three instances; Figure 2B,E). However, *C. auris* isolate 5 exhibited a ZOI to amphotericin B (RAD = 7 mm) and caspofungin (RAD = 6 mm) at 24 h (Figure 2E). As expected, and in agreement with previous work [28], there was an inverse correlation between RAD and MIC (Pearson test, *r* = −0.58, *p* = 0.007).

### 3.3. Identification of Tolerance in C. auris from Broth Microdilution Assays

**Figure 3:**
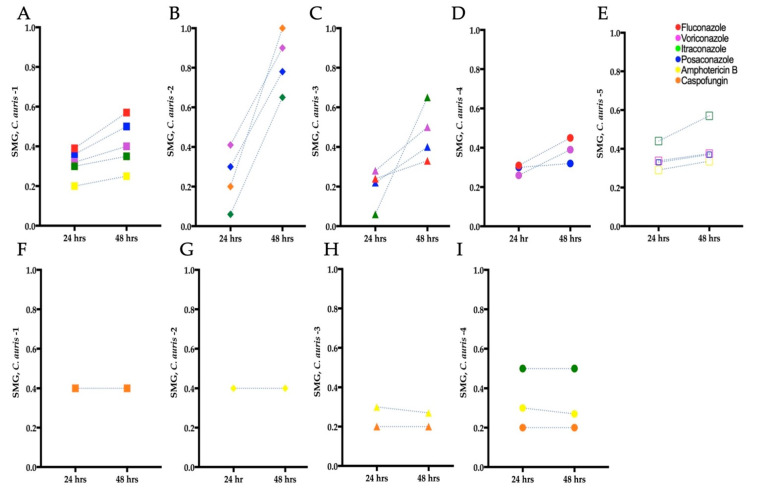
Tolerance from supra-MIC growth (SMG) for clinical *C. auris* isolates grown in antifungal microwell plates. (**A**–**E**) Mean SMG of tolerant isolates after 24 and 48 h. (**F**–**I**) Mean SMG of non-tolerant isolates after 24 and 48 h. *C. auris* 5 was resistant to fluconazole and caspofungin hence tolerance/non-tolerance could not be determined for these isolate–antifungal combinations.

To determine if tolerant subpopulations existed within the non-resistant *C. auris* isolates, we carried out an SMG analysis (Section 2.5). A statistically significant increase in SMG was observed after 48 h for *C. auris* isolate 1 to fluconazole and itraconazole (Independent *t*-test, *p* = 0.009 and *p* = 0.001, respectively), *C. auris* isolates 2 and 4 to voriconazole (Independent *t*-test, *p* = 0.0009 and *p* = 0.0014, respectively), and *C. auris* isolate 2 to caspofungin (Independent *t*-test, *p* = 0.006), indicating the presence of tolerance (Figure 3B,D). There was also a non-significant increase in SMG at 48 h for *C. auris* isolates 3 and 4 to fluconazole, *C. auris* isolates 2, 3, and 5 to itraconazole, *C. auris* isolates 1, 2, 3, 4, and 5 to posaconazole, *C. auris* isolates 1, 3, and 5 to voriconazole, and *C. auris* isolate 1 and 5 to amphotericin B. No tolerance was observed for *C. auris* isolate 4 to itraconazole and caspofungin, and *C. auris* isolates 2, 3, and 4 to amphotericin B (Figure 3F–I). There was a decrease in SMG for *C. auris* isolate 1 against amphotericin B. This occurred because the growth of isolates in wells without antifungals increased over 48 h, which in turn reduced the SMG (as described in Equation (1)). 

### 3.4. Identification of Tolerance in C. auris from Disk Diffusion Assays

**Figure 4:**
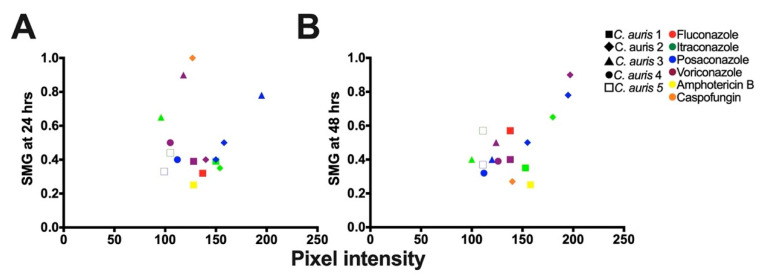
Correlation analysis for mean supra-MIC growth (SMG) and mean pixel intensity measured by ImageJ [51] to determine tolerance. (**A**) Analysis performed after 24 h of growth (R^2^ = 0.3128; Pearson correlation test, *p* = 0.0469). (**B**) Analysis performed after 48 h of growth (R^2^ = 0.2862; Pearson correlation test, *p* = 0.0085).

To confirm the tolerance of the *C. auris* isolates determined by the broth microdilution assays (Section 3.3), we performed the corresponding DDAs. All the *C. auris* isolates with higher SMG exhibited higher FoG_20_ at 48 h (Figure 2F–J). The FoG_20_ within the ZOI ranged between 0.08 and 0.62 and 0.09 and 0.87 at 24 h and 48 h, respectively (Figure 2). *C. auris* isolate 2 exhibited the highest FoG_20_ against caspofungin at 24 h (0.62) and against posaconazole at 48 h (0.87). Similarly, at 24 h the highest pixel intensity occurred for *C. auris* isolate 3 against posaconazole (195, Figure 4A) and the highest SMG occurred for *C. auris* isolate 2 against caspofungin (1.0, Figure 4A). At 48 h, the highest pixel intensity and SMG were measured for *C. auris* isolate 2 against voriconazole (197 and 0.90, respectively; Figure 4B).

There was no correlation between FoG_20_ and RAD levels (Pearson test, *r* = −0.25, *p* = 0.28), as expected based on previous work which established that the FoG_20_ and RAD measure different drug responses [28,29]. The was significant correlation between SMG measured by diskImageR and pixel intensity measured by *ImageJ* (Figure 4), which occurred as both SMG and pixel intensity increase when tolerant subpopulations are present.

Overall, there was no significant difference between diskImageR and manual readings of the RAD (Independent *t*-test, *p* = 0.5634 and *p* = 0.8453 for readings at 24 h and 48 h, respectively; Figure S4). There was also no significant difference for FoG_20_ readings using diskImageR and ImageJ at 24 h (Unpaired *t*-test, *p* = 0.35). However, there was a statistically significant difference for FoG_20_ reading using diskImageR and ImageJ at 48 h (Unpaired *t*-test, *p* = 0.022). The difference in these FoG_20_ readings resulted from the fact that diskImageR was unable to distinguish the border of the ZOI among tolerant isolates, which was obscured by tolerant colonies at 48 h.

Among reference strains, only *C. parapsilosis* exhibited tolerance to fluconazole and voriconazole (Figure S6). The FoG_20_ and SMG for fluconazole and voriconazole is presented in Table S3. No tolerance was observed for the other antifungal drugs considered in this study against *C. parapsilosis*. *I. orientalis* did not exhibit tolerance to any of the antifungal agents tested. 

### 3.5. Tolerance in C. auris Is a Reversable Phenomenon

Next, we investigated if the antifungal tolerance that we discovered in *C. auris* was a reversible phenomenon. To investigate this, we sub-cultured colonies growing inside and outside of the ZOI and repeated the microbroth dilution and disk diffusion experiments (Figure S7). There was no difference between the MICs of original colonies and colonies from inside and outside ZOI at both 24 and 48 h (Table S4). RAD, FoG_20_, and SMG, obtained from *C. auris* colonies isolated from inside and outside the ZOI, also did not show any statistically significant differences. These results indicate that the antifungal-tolerant colonies in our experiments could reversibly generate antifungal-susceptible colonies.

### 3.6. Elimination of Tolerance and Resistance in C. auris via Adjuvant-Antifungal Treatment

To eliminate the tolerance observed in our clinical *C. auris* isolates (Section 3.3 and Section 3.4), a previously known adjuvant chloroquine [37] was combined with the antifungal drugs fluconazole, itraconazole, posaconazole, voriconazole, amphotericin B, and caspofungin. Chloroquine-antifungal disk diffusion assays and broth microdilution assays were performed on all five clinical *C. auris* isolates, as well as on the *C. parapsilosis* and *I. orientalis* reference strains (Table S1). Chloroquine alone did not have any antifungal effect on either *C. auris* isolates or the reference strains (Figure S9).

**Figure 5:**
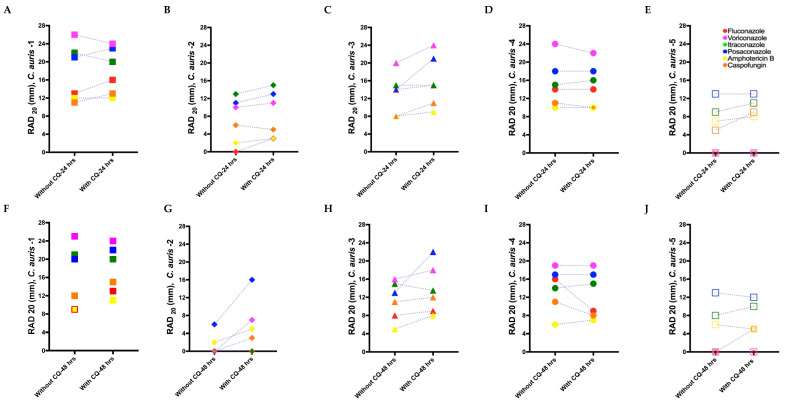

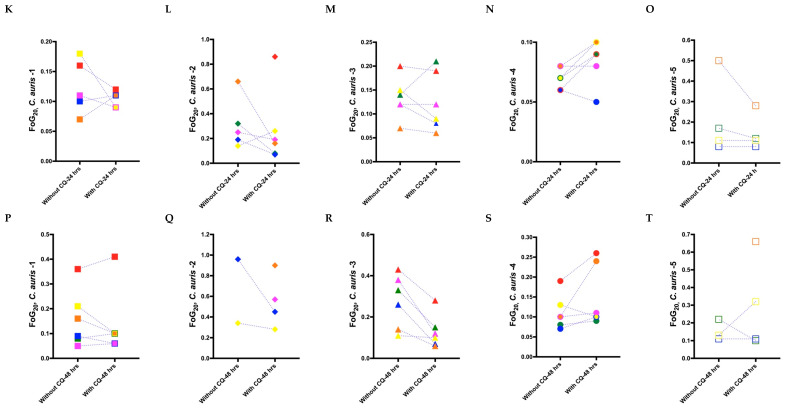
Radius of the zone of inhibition (RAD) and fraction of growth in the zone of inhibition (FoG_20_) measurements for *C. auris* isolates for adjuvant antifungal disk diffusion assays. (**A**–**E**) Mean RAD measured for the *C. auris* isolates at 24 h against antifungal drugs with and without the adjuvant chloroquine. (**F**–**J**) Mean RAD measured for the *C. auris* isolate at 48 h against antifungal drugs with and without chloroquine. (**K**–**O**) Mean FoG_20_ measured using diskImageR [29] for all *C. auris* isolates at 24 h against antifungal drugs with and without chloroquine. (**P**–**T**) Mean FoG_20_ measured using diskImageR for the *C. auris* isolates at 48 h against antifungal drugs with and without chloroquine. Note that the single data points in (**L**), (**Q**), and (**T**) at 48 h are due to the mitigation of resistance in presence of chloroquine, as FoG_20_ could not be measured for these isolates at 24 h because of their resistance to the corresponding antifungal drugs.

Tolerance and resistance were reduced or eliminated in some of our clinical *C. auris* isolates by combing chloroquine with antifungal drugs. *C. auris* isolate 1 showed an increase in RAD for fluconazole, posaconazole, amphotericin B, and caspofungin in presence of chloroquine compared to the RAD measured with these antifungal drugs alone at 48 h (Figure 5A–J). Similar results were found for: *C. auris* isolate 2 for posaconazole, voriconazole, amphotericin B, and caspofungin; *C. auris* 3 for fluconazole, posaconazole, voriconazole, amphotericin B, and caspofungin; *C. auris* isolate 4 for itraconazaole and amphotericin B; and *C. auris* isolate 5 for itraconazole and caspofungin (elimination of resistance for caspofungin), which all displayed an increase in RAD when these antifungal drugs were combined with chloroquine. Correspondingly, the FoG_20_ was reduced in presence of chloroquine for *C. auris* isolate 1 when combined with posaconazole, amphotericin B, and caspofungin (Figure 5K–T). However, no effect was observed when chloroquine was combined with fluconazole, itraconazole, or voriconazole. Similar adjuvant antifungal FoG_20_ results were obtained for *C. auris* isolate 2 against posaconazole, voriconazole, amphotericin B, and caspofungin; *C. auris* isolate 3 against fluconazole, itraconazole, posaconazole, voriconazole, amphotericin B, and caspofungin; *C. auris* isolate 4 against amphotericin B; and *C. auris* isolate 5 against itraconazole and caspofungin. No effect of chloroquine was observed for *C. auris* isolate 4 against fluconazole and posaconazole, nor for *C. auris* isolate 5 against amphotericin B. The FoG_20_ for *C. auris* isolate 2 for voriconazole and caspofungin and *C. auris* isolate 5 for caspofungin could not be measured at 48 h without chloroquine as there was no ZOI. However, we were able to measure the ZOI in some of these isolates in the presence of chloroquine, indicating an adjuvant effect of chloroquine on tolerance as well as on resistance. The reference strain *I. orientalis* (resistant to fluconazole) exhibited a ZOI against fluconazole when supplemented with chloroquine (Table S5; Figure S8). However, *C. parapsilosis* was not significantly affected by the presence of chloroquine (Table S5; Figure S8).

**Figure 6:**
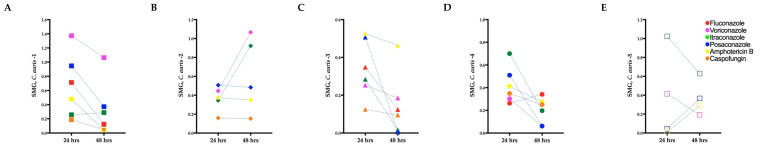
Supra-MIC growth (SMG) of *C. auris* isolates 1 to 5 (**A**–**E**) for adjuvant antifungal broth microdilution assays. Chloroquine did not show any adjuvant effect on C. auris isolate 1 when combined with itraconazole, nor for *C. auris* isolate 2 when combined with itraconazole or voriconazole at 48 h compared to 24 h. Similarly, no adjuvant effect was noted for *C. auris* isolate 4 against fluconazole nor for *C. auris* isolate 5 against posaconazole and amphotericin B. Since *C. auris* isolate 2 is resistant to fluconazole and *C. auris* isolate 5 is resistant to fluconazole and caspofungin, the SMGs were not calculated for these isolate–adjuvant–antifungal combinations.

Similar effects on antifungal tolerance were obtained in adjuvant antifungal broth microdilution assays (Figure 6). Tolerance decreased for all chloroquine–antifungal drug combinations in the following isolates: *C. auris* isolate 1 (except for itraconazole), *C. auris* isolate 2 (except for voriconazole and itraconazole), *C. auris* isolate 3, *C. auris* isolate 4 (except for fluconazole), and *C. auris* isolate 5 (except for posaconazole and amphotericin B) all exhibited reduced SMG with chloroquine–antifungal drug at 48 h compared to SMG at 24 h with chloroquine–antifungal drug. As *C. auris* isolate 2 was resistant to fluconazole, SMG was not calculated. However, chloroquine did not show any effect on *C. auris* isolate-2 against itraconazole and voriconazole at 48 h compared to 24 h which is in concordance with disk diffusion assay. Whereas the SMG for *C. auris* isolate 5 was reduced against itraconazole. However, SMG could not be calculated to fluconazole and caspofungin, due to the growth at highest concentration. Similar to the disk diffusion assays, chloroquine did not show any effect on *C. auris* isolate 1 against itraconazole, *C. auris* isolate 2 against voriconazole and itraconazole, *C. auris* isolate 4 against fluconazole, and *C. auris* isolate-5 against posaconazole and amphotericin B. The MICs of all the *C. auris* isolates and control strains remained unchanged in presence of chloroquine.

## 4. Discussion

We report for the first time that some clinical *C. auris* isolates are tolerant to fungistatic drugs (fluconazole, voriconazole, itraconazole, and posaconazole) and to fungicidal drugs (amphotericin B and caspofungin). We also found azole tolerance in *C. parapsilosis* (fluconazole and voriconazole), but not in *I. orientalis* which was intrinsically resistant to fluconazole. We were able to detect tolerance after 24 h, as well as after 48 h by FoG_20_, of antifungal treatment using diskImageR [29] and ImageJ [52]. These findings suggest that a distinct subpopulation among *C. auris* was able to survive and grow slowly in the presence of different antifungal drugs. Since *C. auris* is a multidrug-resistant pathogen, the presence of tolerance further narrows treatment options. Previous reports suggest that tolerant subpopulations among infecting *Candida* species are strongly associated with mortality among candidemia patients [53]. Therefore, clinical diagnostic laboratories should also test for antifungal tolerance along with standard antifungal susceptibility/resistance tests to increase the efficacy of antifungal treatment. Furthermore, existing tolerance quantification methods could be adapted to detect tolerance after 24 h and 48 h to broaden the scope of standard antimicrobial susceptibility testing in medical diagnostic laboratories. The fluconazole tolerance that we observed in *C. auris* was in agreement with previous studies on *C. albicans* [28] and *C. auris* [23], as well as with related clinical studies on “trailing growth” (reduced but persistent visible growth of *Candida* species in fluconazole concentrations above MIC [32,54,55]).

The tolerance to fungistatic and fungicidal drugs observed in some of the clinical *C. auris* isolates in our study appears to be a reversible phenomenon, as previously described for clinical *C. albicans* isolates [56]. The tolerant cells growing inside ZOI upon subculture are indistinguishable from the parental population, suggesting the presence of phenotypic heterogeneity instead of genetic variation. *C. auris* isolates cultured from inside and outside the ZOI did not show any significant changes in the average RAD, MIC, or SMG levels. This reversible tolerance that we observed in *C. auris* may result from stochastic phenotype switching or an induced response activated by the presence of antifungal drugs inside of the cell. The general mechanism underlying tolerance in *C. auris* remains to be elucidated in future work, to be aided, for instance, by mathematical modeling and synthetic biology [57], tracking single cell growth and gene expression trajectories in microfluidic devices [58,59], as well as genetic sequence and aneuploidy analyses [60].

The tolerance in some of our *C. auris* isolates was reduced or eliminated in vitro by combining azole, polyene, and echinocandin antifungal drugs with the antimalarial drug chloroquine. Chloroquine reduced tolerance for some *C. auris* isolate–antifungal combinations, while chloroquine did not have an adjuvant effect for other combinations. The mechanism underlying this strain-dependent phenomenon remains to be elucidated. Combining chloroquine with antifungal drugs had a partial effect on resistance in some of the *C. auris* isolates investigated in this study. Specifically, *C. auris* isolate 5, which was resistant to caspofungin and voriconazole (RAD = 0 mm), had a small increase in the ZOI (RAD < 12 mm) in presence of chloroquine. Correspondingly, *C. auris* isolate 2 had no ZOI for caspofungin, but had a small ZOI (RAD = 6 mm) in presence of chloroquine. The RADs for these cases were smaller than those for the sensitive *C. auris* isolates in our experiments. Chloroquine did not affect the MICs of the *C. auris* isolates in our study. Chloroquine also affected fluconazole resistance in *I. orientalis* (Table S5), though tolerant subpopulations in *C. parapsilosis* were unaffected by chloroquine. Altogether, these results suggest that combining chloroquine with antifungal drugs may have a partial mitigation effect on resistance in *C. auris*. Though the mechanism of action is unknown, it is likely related to iron depletion caused by chloroquine and its downregulation of the *ERG11* gene [35,41]. Iron depletion is known to decrease membrane sterols and increase membrane fluidity, leading to increased uptake of antifungal drugs into the cell [61]. The downregulation of *ERG11* gene, which synthesizes lanosterol alpha demethylase enzyme, is also known to be an important rate-limiting enzyme for the synthesis of ergosterol [62].

Due to the limited number of *C. auris* isolates that we were able to acquire, the results presented in this study serve as a proof of concept that *C. auris* is tolerant to fungistatic and fungicidal drugs, and that this antifungal tolerance can be mitigated by using chloroquine as an adjuvant. Further in vitro validation of these results in additional *C. auris* isolates, as well as subsequent investigations using in vivo model systems, will be pursued in future research. Another limitation of our study is that we did not have access to patient details and antifungal treatment history due to privacy regulations. 

Overall, this study advances our understanding of antifungal treatment failure in *C. auris* and identifies opportunities for the clinical detection of antifungal tolerance as well as the development of targeted adjuvant antifungal therapies against tolerant and resistant invasive candidiasis.

## Data Availability

The data presented in this study are openly available in Mendeley Data at doi: 10.17632/69zp86jxpz.1.

## Supplementary Materials

The following supporting information can be downloaded at: https://www.mdpi.com/article/10.3390/biomedicines11030898/s1, Table S1: *Candida* isolates and strains; Table S2: minimum inhibitory concentrations (MICs) of *C. auris* isolates; Table S3: mean MIC, SMG, FoG_20_, and RAD for reference strains *Issatchenkia orientalis* and *C. parapsilosis* measured at 24 and 48 h for different antifungal drugs. Table S4: reversibility of tolerance phenotype in *Candida auris*. Table S5: effect of chloroquine (CLQ) on reference strains *Issatchenkia orientalis* and *Candida parapsilosis*. Figure S1: quantification of antifungal tolerance in a disk diffusion assay using the image analysis program diskImageR [29]. Figure S2: detecting tolerance in *Candida auris* from disk diffusion assays (DDAs) using diskImageR. Figure S3: representative disk diffusion assays (DDA) images of fluconazole (FLU) tolerance in *Candida auris* and *Candida parapsilosis*. Figure S4: comparison between diskImageR and manual radius of the zone of inhibition (RAD) measurements. Figure S5: azole tolerance in *Candida auris*. Figure S6: azole tolerance in *Candida parapsilosis* reference strain. Figure S7: reversibility of tolerance in a representative *Candida auris* isolate 2 against voriconazole. Figure S8: disk diffusion assays (DDAs) of antifungal adjuvant treatment in *Candida auris* isolates and *Issatchenkia orientalis* and *Candida parapsilosis* reference strains. Figure S9: *Candida auris* isolates and *Candida parapsilosis* and *Issatchenkia orientalis* reference strains growing on Mueller–Hinton agar (MHA) media with chloroquine.
