# Supplementary material for: Identification and Elimination of Antifungal Tolerance in Candida auris
Source: Biomedicines. 2023 Mar 14;11(3):898. doi: 10.3390/biomedicines11030898 (PMC10045952; doi:10.3390/biomedicines11030898)
Supplement: Supplementary file 1 [file biomedicines-11-00898-s001.zip › biomedicines-2147859-supplementary.pdf]

SUPPLEMENTARY MATERIALS

Supplementary Tables

Table S1. *Candida* isolates and strains. *Candida auris* and *Candida* reference strains *Issatchenkia orientalis* and *Candida parapsilosis* used in our study to investigate antifungal tolerance and resistance. *Issatchenkia orientalis* is also known by the binomial names *Candida krusei* and *Pichia kudriavzevii*.

Strain/Isolate Number	Genus	Species
1	*Candida*	*auris*
2	*Candida*	*auris*
3	*Candida*	*auris*
4	*Candida*	*auris*
5	*Candida*	*auris*
6	*Issatchenkia*	*orientalis*
7	*Candida*	*parapsilosis*

Table S2. Minimum inhibitory concentrations (MICs) of *C. auris* isolates. Mean MICs were measured in μg/mL after 24 and 48 h for a range of fungistatic and fungicidal drugs.

Antifungal drug	Hours of incubation	*C. auris* 1	*C. auris* 2	*C. auris* 3	*C. auris* 4	*C. auris* 5
Fluconazole	24	2	64	2	2	64
	48	2	64	2	2	64
Voriconazole	24	0.03	0.25	0.03	0.03	8
	48	0.03	0.25	0.03	0.03	8
Itraconazole	24	0.03	0.25	0.03	0.03	0.5
	48	0.03	0.25	0.03	0.03	0.5
Posaconazole	24	0.03	0.25	0.03	0.03	0.5
	48	0.03	0.25	0.03	0.03	0.5
Amphotericin B	24	0.5	1	0.5	1	2
	48	1	2	0.5	1	2
Caspofungin	24	1	0.5	0.5	0.5	4
	48	1	0.5	0.5	0.5	4
Anidulafungin	24	0.03	0.25	0.03	0.06	2
	48	0.03	0.25	0.03	0.06	2
Micafungin	24	0.12	0.06	0.06	0.12	0.5
	48	0.12	0.06	0.06	0.06	0.5

Table S3. Mean MIC, SMG, FoG_{20}, and RAD for reference strains *Issatchenkia orientalis* and *C. parapsilosis* measured at 24 and 48 h for different antifungal drugs. MIC: minimum inhibitory concentration; SMG: supra-MIC growth; FoG: fraction of growth; RAD: radius of the zone of inhibition; NA: not available.

		**MIC (µg/mL)**	**SMG at 24 h**	**SMG at 48 h**	**FoG_{20} at 24 h**	**FoG_{20} at 48 h**	**RAD at 24 h (mm)**	**RAD at 48 h (mm)**
I. orientalis ATCC 6258	Fluconazole	32	NA	NA	0	0	0	0
	Itraconazole	0.12	0.62	0.62	0.09	0.09	14	14
	Voriconazole	0.25	0.65	0.25	0.09	0.1	12	12
	Posaconazole	0.06	0.16	0.5	0.09	0.11	12	12
	Amphotericin B	1	0.31	0.25	0.2	0.06	6	3
	Caspofungin	0.5	0.62	0.5	0.13	0.22	11	11
C. parapsilosis ATCC 22019	Fluconazole	2	0.41	0.47	0.13	0.13	15	13
	Itraconazole	0.03	0.53	0.45	0.1	0.1	14	14
	Voriconazole	0.03	0.34	0.66	0.13	0.16	18	18
	Posaconazole	0.03	0.5	0.5	0.08	0.07	16	16
	Amphotericin B	0.5	0.24	0.24	0.17	0.17	9	8
	Caspofungin	1	0.76	0.1	0.19	0.18	7	6

Table S4. Reversibility of tolerance phenotype among tolerant *Candida auris* isolates against different antifungal agents. Mean radius of the zone of inhibition (RAD), mean fraction of growth (FoG) in the zone of inhibition (ZOI), mean minimum inhibitory concentration (MIC), and mean supra-MIC growth (SMG) values obtained for *C. auris* isolates sub-cultured from inside and outside the ZOI, and treated with the azole, polyene, and echinocandin antifungal drugs. MIC, RAD, FoG_{20}, and SMG, obtained from *C. auris* colonies isolated from inside and outside the ZOI, also did not show any statistically significant differences (Independent t-test, $p > 0.05$ for all values).

Drug–isolate combination	Origin of the colonies tested	MIC in µg/ml	RAD_{20} at 24 h (mm)	RAD_{20} at 48 h (mm)	FoG_{20} at 24 h	FoG_{20} at 48 h	SMG at 24 h	SMG at 48 h
Fluconazole-*C. auris* 1	Original	2	13	10	0.14	0.21	0.38	0.57
	Inside ZOI	2	15	7	0.13	0.22	0.54	0.66
	Outside ZOI	2	14	8	0.15	0.43	0.62	0.64
Fluconazole- *C. auris* 3	Original	2	14	8	0.11	0.29	0.26	0.33
	Inside ZOI	2	10	10	0.20	0.83	0.20	0.77
	Outside ZOI	2	12	9	0.21	0.71	0.50	0.78
Fluconazole-*C. auris* 4	Original	2	14	10	0.12	0.24	0.30	0.45
	Inside ZOI	2	16	13	0.19	0.34	0.33	0.40
	Outside ZOI	2	13	11	0.08	0.20	0.46	0.55
Itraconazole-*C. auris* 1	Original	0.03	17	17	0.09	0.12	0.30	0.50
	Inside ZOI	0.03	17	17	0.06	0.09	0.26	0.48
	Outside ZOI	0.03	17	17	0.09	0.09	0.29	0.50
Itraconazole-*C. auris* 2	Original	0.25	10	0	0.09	NA	0.06	0.65
	Inside ZOI	0.25	15	0	0.13	NA	0.05	0.60
	Outside ZOI	0.25	15	0	0.09	NA	0.06	0.80
Itraconazole-*C. auris* 3	Original	0.03	11	0	0.11	NA	0.22	0.40
	Inside ZOI	0.03	12	0	0.08	NA	0.32	0.45
	Outside ZOI	0.03	15	0	0.14	NA	0.40	0.59
Itraconazole-*C. auris* 5	Original	0.5	16	0	0.13	0.11	0.44	0.57
	Inside ZOI	0.5	8	6	0.12	0.22	0.40	0.44
	Outside ZOI	0.5	9	8	0.11	0.25	0.58	0.61
Voriconazole-*C. auris 1*	Original	0.03	20	20	0.07	0.10	0.32	0.40
	Inside ZOI	0.03	23	18	0.11	0.18	0.37	0.40
	Outside ZOI	0.03	20	10	0.12	0.10	0.43	0.44
Voriconazole-*C. auris* 2	Original	0.25	9	0	0.15	NA	0.41	0.9
	Inside ZOI	0.25	11	0	0.20	NA	0.27	1.0
	Outside ZOI	0.25	10	0	0.31	NA	0.26	1.3
Voriconazole-*C. auris* 3	Original	0.03	23	17	0.06	0.25	0.28	0.50
	Inside ZOI	0.03	19	14	0.10	0.36	0.28	0.51
	Outside ZOI	0.03	19	17	0.11	0.38	0.30	0.60
Voriconazole-*C. auris* 4	Original	0.03	21	18	0.07	0.12	0.26	0.39
	Inside ZOI	0.03	21	19	0.09	0.12	0.21	0.28
	Outside ZOI	0.03	23	23	0.08	0.09	0.32	0.35
Posaconazole-*C. auris* 1	Original	0.03	19	19	0.09	0.08	0.36	0.50
	Inside ZOI	0.03	19	21	0.05	0.08	0.34	0.64
	Outside ZOI	0.03	20	20	0.10	0.07	0.32	0.67
Posaconazole-*C. auris* 2	Original	0.25	13	7	0.07	0.80	0.30	0.78
	Inside ZOI	0.25	14	8	0.09	0.64	0.28	0.67
	Outside ZOI	0.25	14	7	0.10	0.70	0.28	0.85

Posaconazole-C. *auris* 3	Original	0.03	16	15	0.07	0.16	0.22	0.40
	Inside ZOI	0.03	18	15	0.07	0.26	0.24	0.70
	Outside ZOI	0.03	17	18	0.07	0.19	0.25	0.87
Posaconazole-C. *auris* 4	Original	0.03	13	14	0.07	0.12	0.30	0.32
	Inside ZOI	0.03	20	20	0.07	0.09	0.30	0.42
	Outside ZOI	0.03	20	21	0.1	0.08	0.25	0.36
Posaconazole-C. *auris* 5	Original	0.5	12	12	0.07	0.09	0.33	0.37
	Inside ZOI	0.5	12	11	0.14	0.12	0.23	0.32
	Outside ZOI	0.5	11	10	0.12	0.12	0.30	0.35
Amphotericin B- *C. auris* 1	Original	0.5	11	12	0.13	0.15	0.2	0.25
	Inside ZOI	0.5	7	6	0.12	0.15	0.2	0.6
	Outside ZOI	0.5	10	7	0.11	0.2	0.2	0.53
Caspofungin- *C. auris* 2	Original	0.5	8	0	0.22	NA	0.2	1
	Inside ZOI	0.5	8	0	0.26	NA	0.30	1
	Outside ZOI	0.5	8	0	0.25	NA	0.29	1

Table S5. Effect of chloroquine (CLQ) on *Issatchenkia orientalis* and *Candida parapsilosis* reference strains. Mean FoG_{20}: fraction of growth; Mean RAD: radius of the zone of inhibition. The *p*-value was obtained by comparing RAD at 48 h with and without chloroquine.

		**Without CLQ**	**With CLQ**	**Without CLQ**	**With CLQ**	**Paired t-test**
		FoG_{20} at 48 h	FoG_{20} at 48 h	RAD (mm) at 48 h	RAD (mm) at 48 h	
C. parapsilosis ATCC 22019	Fluconazole	0.14	0.08	14	9	*p* = 0.195
	Posaconazole	0.07	0.06	17	16	*p* = 0.272
I. orientalis ATCC 6258	Fluconazole	NA	0.11	0	7	*p* = 0.032

Supplementary Figures

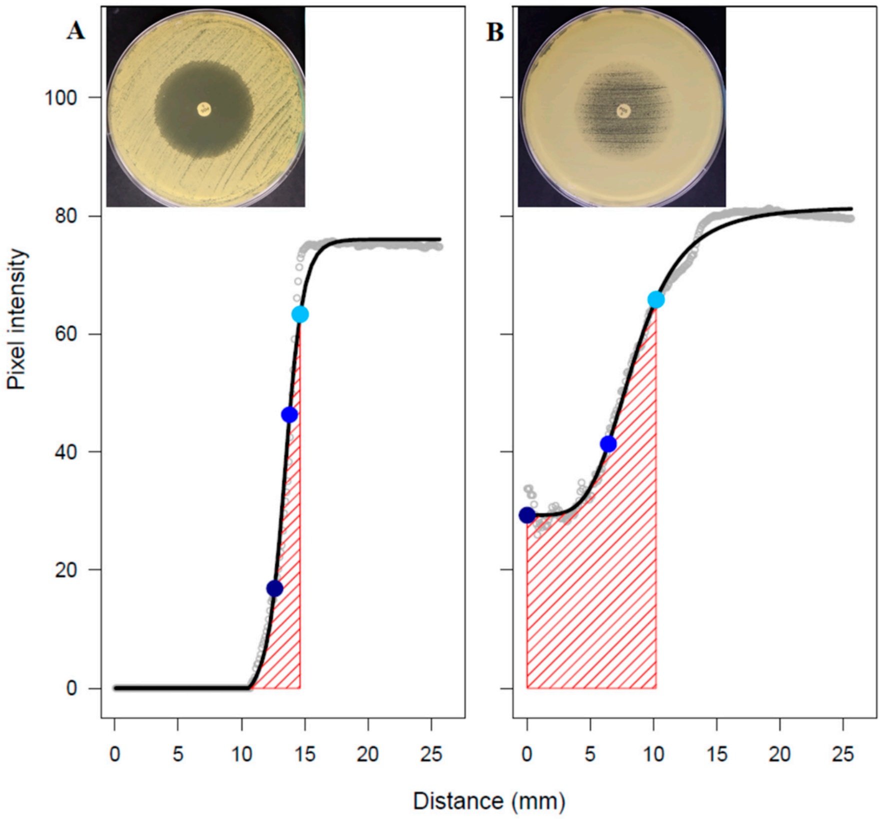

Figure S1. Quantification of antifungal tolerance in a disk diffusion assay using the image analysis program diskImageR [29]. Pixel intensity corresponds to the cell density, and its average is measured for 72 radii every 5 ° from the center of the disk (grey dots). The radius of the zone of inhibition and fraction of growth are measured in three areas where 20%, 50%, and 80% of the growth is inhibited (light blue, blue, and dark blue circles, respectively) after (A) 24 h of incubation and (B) 48 h of incubation. The representative data in this figure was obtained from images of a disk diffusion assays for *C. auris* (isolate 2) exposed to posaconazole (insets of (A) and (B)).

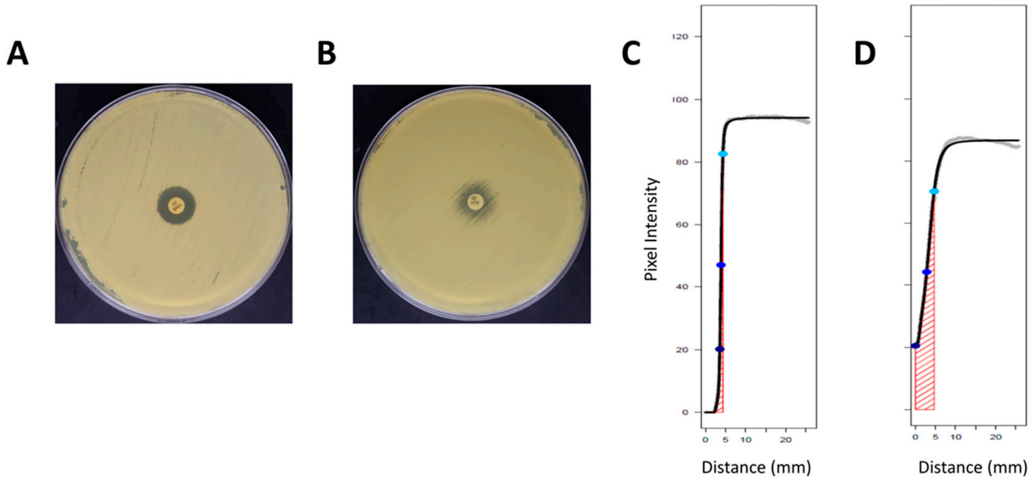

Figure S2. Detecting tolerance in *Candida auris* from disk diffusion assays (DDAs) using *diskImageR*. **(A)** Representative DDA image of *C. auris* (isolate 1) after 24 h of exposure to amphotericin B (AMB). **(B)** Representative DDA image of *C. auris* (isolate 1) after 24 h of exposure to fluconazole (FLU). **(C)** Quantification tolerance (shown in the pink zone) from the DDA shown to FLU in (A) using *diskImageR* [29]. **(D)** Quantification tolerance from the DDA shown to AMB in (B) using *diskimageR*.

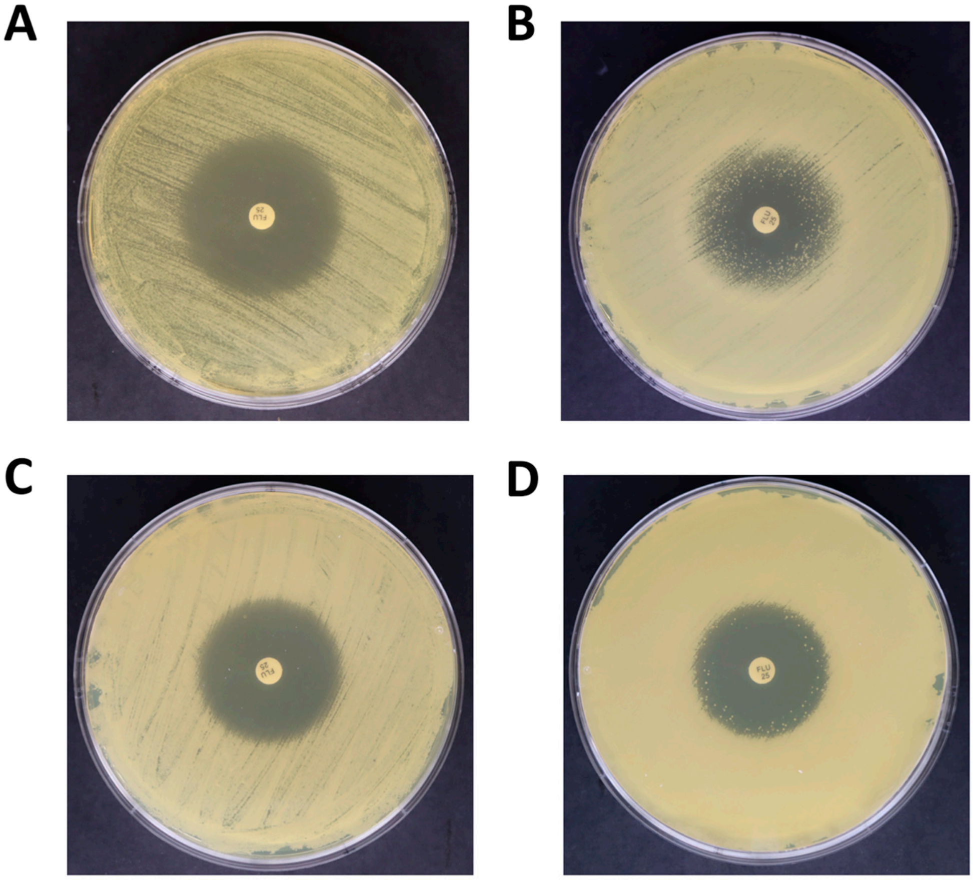

Figure S3. Representative disk diffusion assays (DDA) images of fluconazole (FLU) tolerance in *Candida auris* and *Candida parapsilosis*. **(A)** DDA of *C. auris* (isolate 1) after 24 h of exposure to FLU. **(B)** DDA of *C. auris* (isolate 1) after 48 h of exposure to FLU. **(C)** DDA of *C. parapsilosis* after 24 h of exposure to FLU. **(D)** DDA of *C. parapsilosis* after 48 h of exposure to FLU.

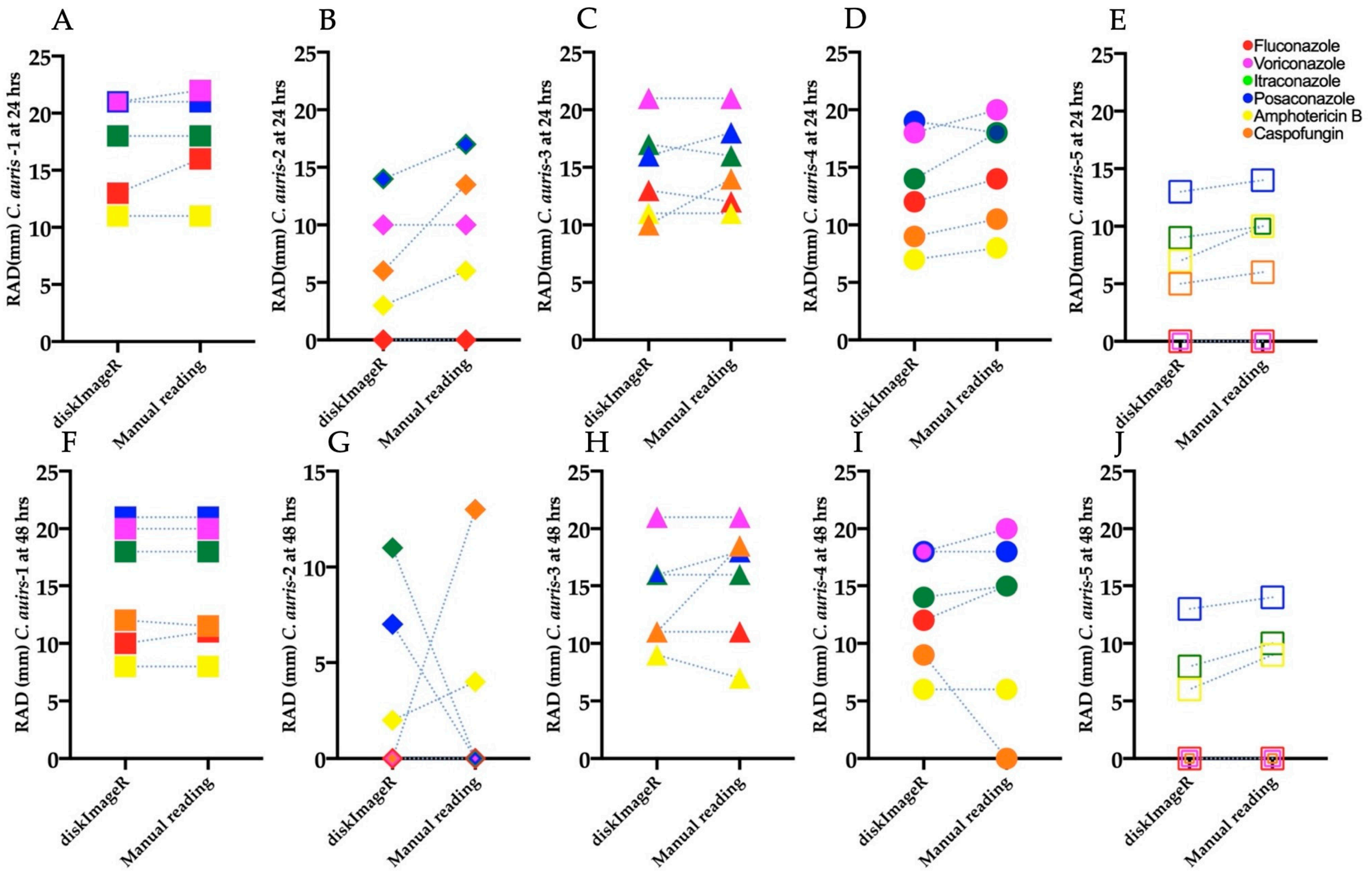

Figure S4. Comparison between *diskImageR* and manual radius of the zone of inhibition (RAD) measurements. **(A-E)** Mean RAD: radius of the zone of inhibition measured by *diskImageR* [29], and manually (see Section 2.5) at 24 h and **(F-J)** at 48 h.

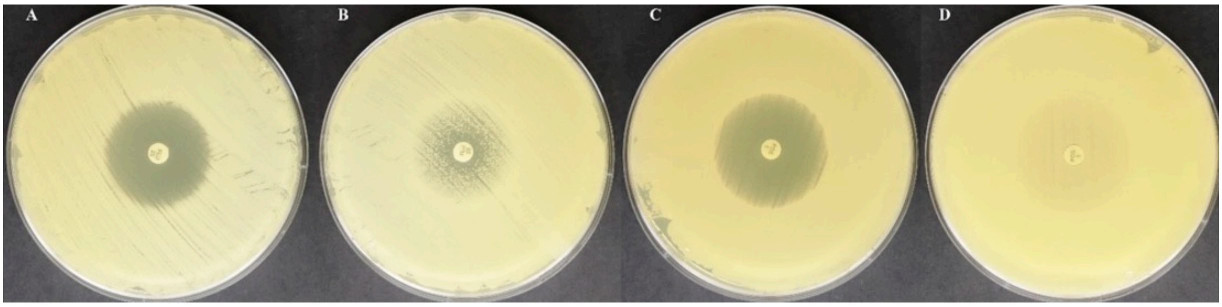

Figure S5. Azole tolerance in *Candida auris*. Disk diffusion assay (DDA) of fluconazole for *C. auris* (isolate 1) after **(A)** 24 h of growth and **(B)** 48 h of growth. DDA of posaconazole for *C. auris* isolate 2 after **(C)** 24 h of growth and **(D)** 48 h of growth.

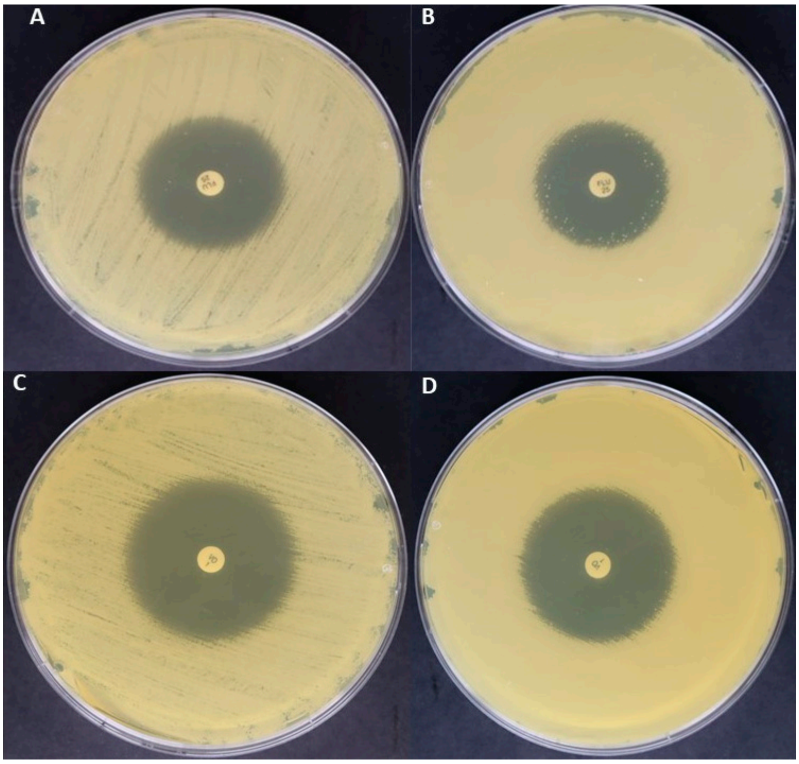

Figure S6. Azole tolerance in the *Candida parapsilosis* reference strain. Tolerant *C. parapsilosis* colonies in the zone of inhibition (ZOI) after (A) 24 h and (B) 48 h of fluconazole treatment. Tolerant *C. parapsilosis* colonies in the ZOI after (C) 24 h and (D) 48 h of voriconazole treatment.

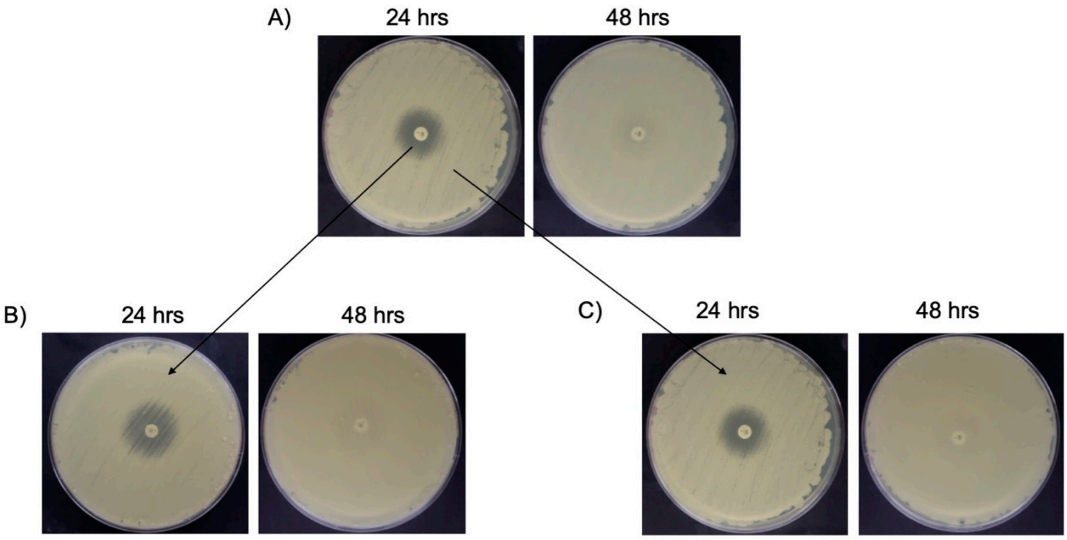

Figure S7. Reversibility of tolerance in a representative *Candida auris* isolate 2 against voriconazole. Disk diffusion assays after 24 and 48 h for (A) *C. auris* original isolate 2, (B) colonies isolated and sub-cultured from inside the zone of inhibition (ZOI) of the original plate, and (C) colonies isolated and sub-cultured from outside the ZOI of the original plate.

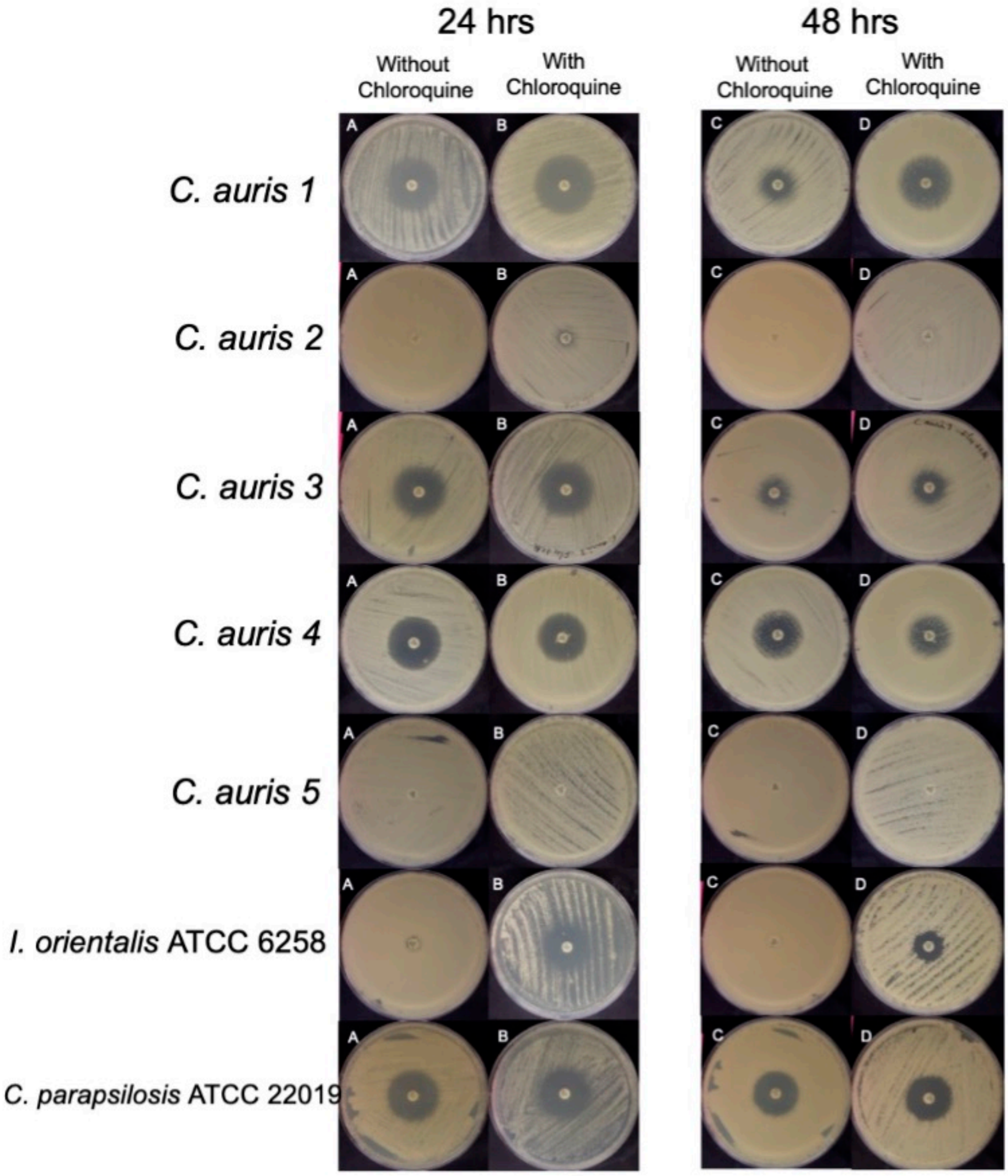

Figure S8. Disk diffusion assays (DDAs) of antifungal adjuvant treatment in *Candida auris* isolates and *Issatchenkia orientalis* and *Candida parapsilosis* reference strains. DDAs with fluconazole (FLU; 1st column) and with FLU combined with chloroquine (2nd column) against five *C. auris* isolates and two reference strains after 24 h (left) and 48 h (right).

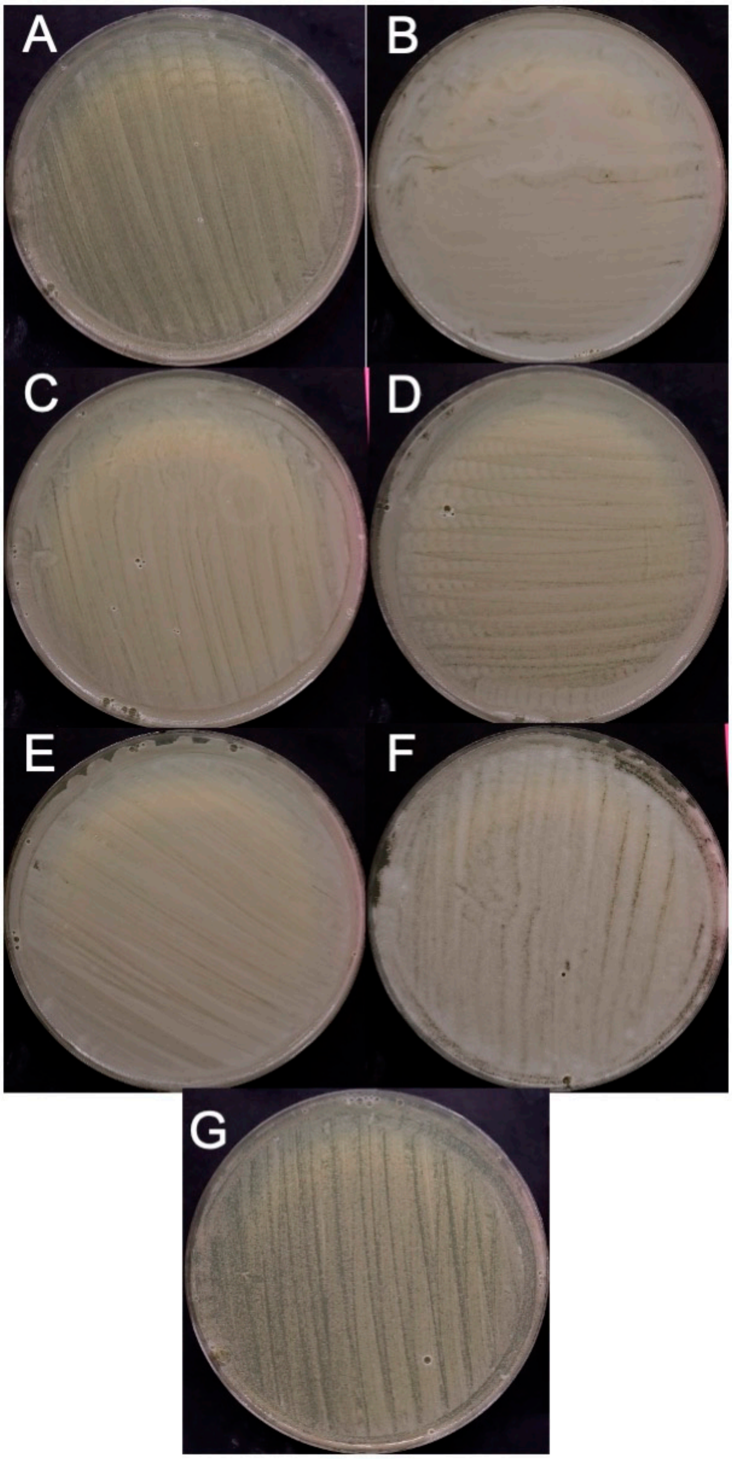

Figure S9. *Candida auris* isolates and *Candida parapsilosis and Issatchenkia orientalis* reference strains growing on Mueller–Hinton agar (MHA) media with chloroquine. Images of *C. auris* isolates 1-5 (A-E), *I. orientalis* (F), and *C. parapsilosis* (G) grown on MHA plus glucose methylene blue agar plates with 1031.8 µg/mL chloroquine diphosphate salt.
